# Network Pharmacology Approach Reveals the Potential Immune Function Activation and Tumor Cell Apoptosis Promotion of Xia Qi Decoction in Lung Cancer

**DOI:** 10.3390/medsci8010001

**Published:** 2019-12-29

**Authors:** Song Zhang, Yun Wang

**Affiliations:** School of Chinese Materia Medica, Beijing University of Chinese Medicine, Beijing 10029, China; zhangs@bucm.edu.cn

**Keywords:** traditional Chinese medicine, Xia Qi Decoction, network pharmacology, lung cancer, immunocyte, apoptosis of tumor cells

## Abstract

As the leading cause of cancer death worldwide, lung cancer (LC) has seriously affected human health and longevity. Chinese medicine is a complex system guided by traditional Chinese medicine theories (TCM). Nowadays, the clinical application of TCM for LC patients has become the focus for its effectiveness and security. In this paper, we will analyze and study the mechanism of Xia Qi Decoction (XQD) in the treatment of LC. The results collectively show that XQD could act on 41 therapeutic targets of LC. At the same time, 8 of 41 targets were significantly expressed in immune tissues and cells by activating CD8+T cells to promote apoptosis of cancer cells. It reveals the molecular mechanism of XQD in the treatment of LC from the perspective of network pharmacology. In addition, in the treatment of LC, XQD can activate (up-regulate) the function of immune cells, promote the apoptosis of tumor cells, and have an active anti-tumor immune effect. In conclusion, this study reveals the unique advantages of traditional Chinese medicine in the treatment of cancer, in reinforcing the healthy qi and eliminating the pathogenic factors. More research, however, is needed to verify the potential mechanisms.

## 1. Introduction

Lung cancer (LC), as the second most common cancer in the world, has the highest mortality rate. Specific biomarkers for its diagnostics, treatment, and prognosis are still under further investigation [1], and the incidence of LC continues to increase in China [2,3]. Concurrently, LC is the leading cause of cancer death in China [4]. Although much research effort has been made in improving treatment for LC in recent decades, a survival rate of five years is still less than 20% [5]. There are two main pathological types of LC: small cell lung cancer (SCLC) accounting for about 85% and non-small cell lung cancer (NSCLC) accounting for about 13%–15% [6]. The main symptoms of LC are coughing, hemoptysis, dyspnea, and fatigue. Traditional Chinese medicine (TCM), as an important part of the healthcare system in China, has gradually gained popularity both at home and abroad [7]. There is a general consensus that TCM produces therapeutic effects in a holistic manner [8]; besides, multi-target therapy is more effective than single therapy in combating polygenic diseases [9].

With the development of systems biology, network biology, and pharmacology, Andrew L. Hopkins proposed the concept of network pharmacology [10]. Network pharmacology studies the treatment or influence of drugs on diseases from the network level, and reveals the synergism law of multi-component drugs, so as to discover the high efficiency and low toxicity of multi-target drugs. Therefore, from the molecular level, the idea of TCM is consistent with those of network pharmacology [11].

In recent years, TCMs have been widely applied in the treatment of cancers in China and beyond. XQD, as a classic herbal formula, was created by Huang Yuanyu in the Qing Dynasty from “Si Sheng Xin Yuan”. XQD is mainly used to treat diseases of lung and stomach system. XQD is composed of eight medicinal herbs: Licorice (LO), Schisandra chinensis (SC), Poria cocos (PC), Pinellia ternata (Thunb) Breit (AT), Citrus reticulata (CR), Fritillaria cirrhosa D. Don (FR),Paeoniae radix alba (PR), and Amygdalus communis vas (AC). XQD is commonly used to treat lung diseases such as cough, asthma, and lung cancer [12,13]. Clinical experiments found that Xiaqi Decoction can also treat the acute exacerbation of chronic bronchitis [14]. Furthermore, it was found that the total effective rate of the experimental group was 86.7%, which was significantly higher than that of the control group (63.3%), after clinical comparison experiments [15]. However, the effective compounds, targets and pharmacological mechanisms of XQD in the treatment of lung cancer remained unclear. Therefore, in this paper, a network pharmacology approach, functional gene pathway analysis, network analysis, and other comprehensive methods were used to reveal XQD-related active compounds, key therapeutic targets, and the molecular mechanism of action for LC.

## 2. Materials and Methods

### 2.1. Collection of XQD Chemical Ingredients

TCMSP database (http://sm.nwsuaf.edu.cn/lsp/tcmsp.php.) is a traditional Chinese medicine systems pharmacology database and analysis platform [16]. To determine the chemical ingredients of the eight herbs contained in XQD, we performed a search by TCMSP. In order to maximize the chances of finding the fully active compounds, we set two conditions as the criteria for screening these active compounds: oral bioavailability (OB) and drug-likeness (DL), which are the two most important indicators of evaluating ADME characteristics via bioinformatics.

### 2.2. Target Selection and Herb-Ingredient-Target Network Construction of XQD

After discovering 112 active ingredients of eight traditional Chinese medicines of XQD, the next important step was to identify their molecular targets and trigger a series of biological effects. The potential targets of XQD were also identified from DrugBank database [17] and the database of our research group [18]. Herb-ingredient-target network with Cytoscape3.7 software was constructed. Nodes in the network were herbs, ingredients and targets. Edges were used to connect herbs with ingredients, active ingredients, and act targets to show the relationship between herbs, active ingredients, and targets in order to explore the multiple pharmacological mechanisms of XQD based on the constructed network.

### 2.3. Collection of Therapeutic Targets for LC

Therapeutic targets for LC were collected from the following four databases: NCBI database [19], TTD database [20], OMIM database [21], and DrugBank database [17]. Using “Lung cancer” to search in the four databases mentioned above, the duplicate targets were eliminated, and 228 targets related to human LC were finally obtained.

### 2.4. Protein-Protein Interaction Data

Protein–protein interaction (PPI) data were from STRING database [22] and an auxiliary elucidation system for the TCM mechanism of our team laboratory, which realized the automatic establishment of biological network model [18]. Entity grammar systems is a formal grammar system [23], which is extended to Chomsky generative grammar system and has been used for the analysis of multiple mechanisms of action of traditional Chinese medicine. It has the characteristics of high efficiency and flexibility, and is suitable for the study of complex biological systems. The ID type of protein is converted to UniProt ID [24].

### 2.5. Herb-Ingredient-LC Therapeutic Target Network Analysis

The ingredients-target network was mapped to LC-related gene network. Furthermore the interaction network between the target of XQD and LC-related genes was established. If the chemical components of XQD overlap with the therapeutic targets of LC, then these targets are the direct targets of XQD for LC treatment. If the chemical targets of XQD act on the therapeutic targets of LC through one or two protein interactions, then these targets are indirect targets of XQD for LC. The direct and indirect targets of XQD on LC were found, and the mechanism was determined. In this network, the nodes represent Chinese medicine, ingredients, targets or genes and LC, and the links between these nodes. “Degree value” is the number of edges associated with it. Targets with connectivity greater than twice the average number of node degrees are selected as key nodes in the network [25].

### 2.6. Pathway Enrichment Analysis

KEGG (Kyoto Encyclopedia of Genes and Genomes) is a database of genome decipherment. The KEGG pathway was used to analyze the main pathways of XQD acting on 41 targets of LC. ClueGO: a Cytoscape plug-in to decipher functionally grouped gene ontology and pathway annotation networks [26], which was used to integrate the KEGG pathways [27].

### 2.7. Relationship Analysis Between LC Targets and Immunological Targets Affected by XQD

41 genes of LC affected by XQD were searched in GeneCards database [28] to find the expression of genes (targets) in human immune tissues and cells. Chi-square test was used to screen the genes significantly expressed in immune tissues and cells. We constructed a network between the selected genes and immune tissues and cells, to explore the mechanism of the key genes regulating LC and immunity.

## 3. Results

### 3.1. OB Prediction and DL Calculation

According to TCMSP database of TCM systematic pharmacology and analysis platform, the chemical ingredients of the eight traditional Chinese medicines of XQD were identified. In order to obtain the potential active ingredients of XQD, the chemical were evaluated and screened using OB ≥ 30% [29], DL ≥ 0.18 [30], respectively. The chemical ingredients of eight traditional Chinese medicines in XQD were as follows: 92 in LO,13 in AT,15 in PC,19 in AC,13 in FR,8 in SC,13 in PR,5 in CR. In order to further screen the active ingredients of LO, the OB of which was increased to more than 50%. Eventually, 112 active ingredients were screened out (Table 1).

### 3.2. Target Selection and Herb-Ingredient-Target Network Construction of XQD

**Figure 1:**
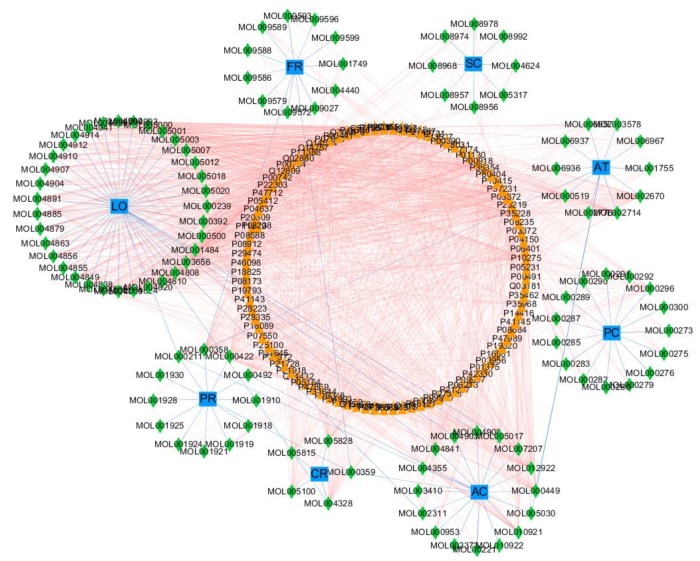
Herb-ingredient-target network. Blue square: herbs, green diamond: ingredients, orange triangle: targets, Blue edges: the relationship between herbs and components; Pink edges: the relationship components herbs and targets.

Ninety-six potential targets were obtained from TCMSP and our laboratory database. Cytoscape software was used to construct a network of herb-ingredient-target. The nodes in the network were Chinese herbs, components, and targets; in addition, the edge of the network represented the relationship between herb–component and component–target. By introducing 8 herbs, 112 ingredients, and 96 targets into Cytoscape, three kinds of nodes were connected to construct a network of traditional Chinese medicines, active ingredients, and targets, as shown in Figure 1.

The network consists of 8 herbs, 112 chemical ingredients, 96 targets, 234 nodes, and 1498 edges. The Centiscape2.2 plugin is used to screen the key nodes in the network. Centrality is used to screen the targets. Using Centiscape2.2, we can calculate Degree Centrality (DC), Closeness Centrality (CC), and Betweenness Centrality (BC). Degree value denotes the number of routes connected to the node in the network. In this network, Degree = 13.833, Betweenness Centrality = 0.008, Closeness Centrality = 0.365. Among 50 of 112 active ingredients are active ingredients with degree greater than 14 and degree of 9 active ingredients is greater than twice the average degree in Table 2. Among the 96 targets, degree of 29 targets is more than 14 and degree of 19 targets is more than twice the average in Table 3. Therefore, these 19 targets are very likely to be the key targets for XQD to play a therapeutic role.

In the herb-ingredient-target network, 45.5% of the chemical ingredients had more than 14 targets, and 12 of them had more than 28 targets in Table 3. This indicated that most of the chemical ingredients in XQD could simultaneously act on multiple targets to play a combined therapeutic role. For example, Beta-sitosterol, a chemical ingredients contained in Fritillaria chuanxiong, AT, and PR, has the most targets and can interact with 49 targets. Secondly, the number of targets of Kaempferol in PR and L-SPD in AC was 44. Beta-sitosterol is a plant derived nutrient with anticancer properties against LC, stomach cancer, ovarian cancer, and leukemia. Studies have shown that BS interfere with multiple cell signaling pathways, including cell cycle, apoptosis, survival, invasion, angiogenesis, and metastasis [31]. Kaempferol is the most common flavonoid compound, which has inhibitory effects on LC [32], ovarian cancer [33], breast cancer [34], and many other tumors.

In the network, the highest degree target was AR, corresponding to 87 chemical compounds. Positive expression of AR might be correlated with the progression and the lymph node metastasis of lung cancer [35]. AR could inhibit the proliferation and survival of cancer cells by up-regulating PTEN directly [36]. Secondly, ESR1 had 74 chemical ingredients that can act on this target. In non-small cell lung cancer, ESR1 can be combined with EGFR, showing enhanced antiproliferation effects [37]. Therefore, it was speculated that these key components and targets were closely related to the mechanism of XQD in the treatment of LC.

### 3.3. Construction of PPI Network in XQD

**Figure 2:**
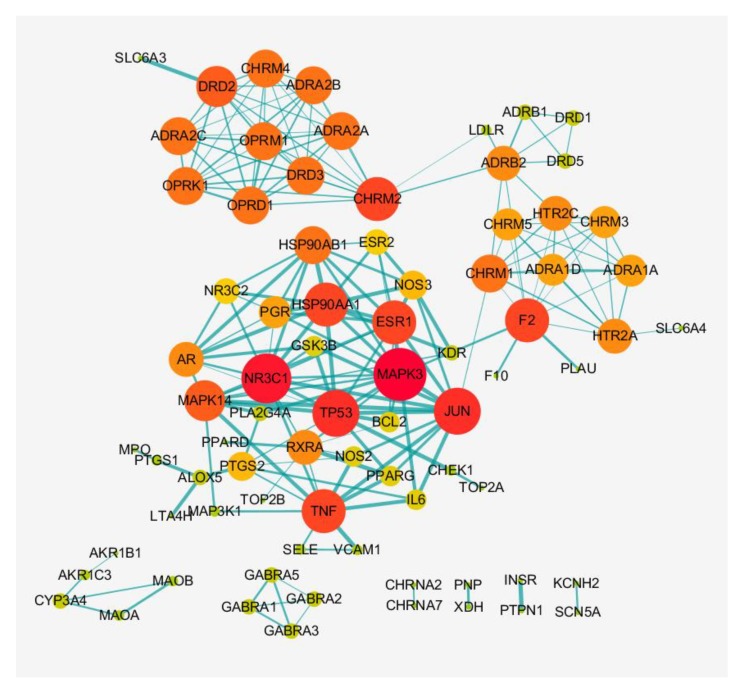
The protein–protein interaction (PPI) of 82 targets.

96 targets of XQD were imported into STRIING database, and the species were limited to human beings. Some proteins did not interact with each other, which were not reflected in the interaction network. The highest confidence level with score greater than 0.9 was selected to obtain the protein network. Average degree value = 5.15, and 13 non-interactive targets were excluded. In PPI networks with 82 targets, the size and color of the target were set to reflect the Degree size, the minimum value of Degree = 1, and the maximum value of Degree = 14. The thickness of the table was set to reflect the combine size in Figure 2.

The PPI network (Figure 2) of 82 targets included 82 nodes and 207 edges. Eight targets with a degree greater than twice the median were MAPK3 (Degree = 14), JUN (Degree = 12), TP53 (Degree = 12), HSP90AA1 (Degree = 11), TNF (Degree = 11), ESR1 (Degree = 11), F2 (Degree = 11), CHRM2 (Degree = 11). A total of 8 central targets were obtained. These eight targets play a key role in the protein network and become the hub connecting other nodes in the network. Among them, the degree of MAPK3 (mitogen-activated protein kinase 3) was the highest (Degree = 14). This indicates that these targets play a key role in the network and become the hub connecting other targets in the network.

### 3.4. Constructing the Network of Herb-Ingredient-Target-LC Therapeutic Target

**Figure 3:**
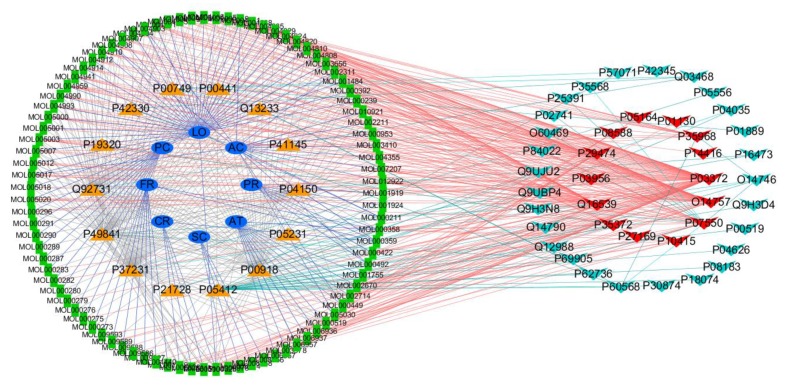
The shapes of different colors represent different types of nodes. Blue ellipse represents traditional Chinese medicine, green square represents chemical composition, orange triangle represents the predictive target of XQD, red triangle represents the direct target of XQD for lung cancer treatment, and light blue represents the indirect target of XQD for LC treatment.

To construct the network of XQD and lung cancer treatment, 91 of the 112 chemical ingredients of XQD act on 41 targets of LC through direct or indirect protein interaction. Degree = 8.09, Betweenness centrality = 0.012, Closeness centrality = 0.356. Ultimately, as shown in Figure 3, the degree value of 25 components was more than 8. There are 25 key components whose degree value is greater than the average degree value (Table 4). These components could be considered as the key compounds of XQD in the treatment of LC. In the network (Figure 3), we screened 14 direct targets (Table 5) and 27 indirect targets (Table 6) for LC. 

They were mainly: Beta-sitosterol (degree = 17) in PR, AT and FR can act on 14 targets of LC. Kaempferol in PR (degree = 14) acts on 13 targets of LC. Beta-sitosterol is present in PR, having anti-tumor, anti-microbial, and immunomodulatory activities [38]. In vitro studies showed that Beta-sitosterol increased the number of viable peripheral blood mononuclear cell (PBMC) and activated swine dendritic cells (DCs) in culture [39]. Beta-sitosterol (β-sitosterol) induced G0/G1 cell cycle arrest and inhibited cell proliferation in A549 cells. These results indicate that beta-sitosterol may serve as novel targets for the treatment of NSCLC [40]. Bio-assay guided fractionation showed the presence of phytosteols (β-sitosterol) which significantly inhibited the growth of A549 cells and promoted apoptosis alone or in combination. This study ensures that these phytosterols, alone or in combination, can be considered as safe and potential drug candidates for LC treatment [41]. Kaempferol in PR can act on 13 targets of LC. MEK-MAPK is a requirement for kaempferol-induced cell death machinery in A549 cells [32]. Cavidine (degree = 13) in AT acts on 12 targets of LC. Cavidine exists in AT and has significant anti-inflammatory effect, which inhibits the production of proinflammatory cytokines TNF-alpha and IL-6 [42]. At the same time, Cavidine has anti-inflammatory activity to prevent inflammatory injury induced by lipopolysaccharide (LPS) [42,43].

Naringenin (degree = 12) in LO and formononetin (degree = 11) in AC can act on 10 targets of LC, respectively. The cumulative effect of these four ingredients on the number of therapeutic targets for LC is 22. Studies have shown that Naringenin, a natural product that is mainly present in LO, may contribute to cancer prevention. There are many advantages compared to traditional chemotherapeutic drugs, such as low toxicity, which can also inhibit the number of lung cancer cells metastasis by regulating immunity [44]. Thus, it has a potential to inhibit lung cancer [44,45]. Naringenin up-regulates the expression of death receptor 5 and enhances TRAIL-induced apoptosis in human lung cancer A549 cells, with no detectable inhibitory effects on cell proliferation of normal lung fibroblast cells [46]. Formononetin in AC was investigated the anti-proliferative effects on human non-small cell lung cancer (NSCLC). It inhibits proliferation of two NSCLC cell lines (A549 and nci-h23), induces G1 phase cell cycle arrest, and promotes NSCLC cell apoptosis. The results demonstrated that formononetin might be a potential chemopreventive drug for lung cancer therapy through induction of cell cycle arrest and apoptosis in NSCLC cells [47]. Baicalein (degree = 10) in AT is a widely used Chinese herbal medicine, traditionally used as anti-inflammatory and anti-cancer therapy. Baicalein significantly decreased lung cancer proliferation in H-460 cells in a dose-dependent induction in apoptosis. This was the first time that baicalin had been effective in vitro and in vivo in NSCLC [48]. Experimental studies had shown that Baicalein induced cell cycle arrest and apoptosis in human lung squamous carcinoma CH27 cells [49].

### 3.5. Pathway Analysis of XQD Acting on LC Therapeutic Targets

**Figure 4:**
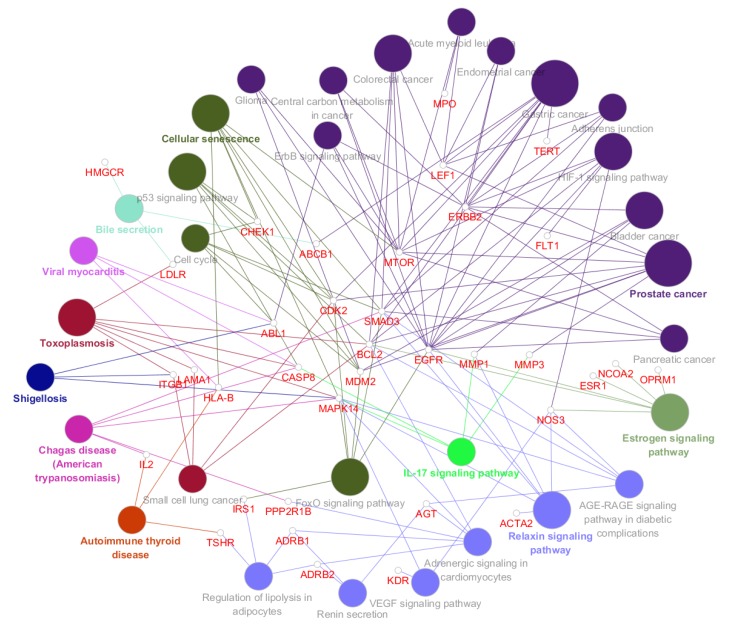
Target-pathway network. Small nodes stand for therapeutic targets for lung cancer. Large nodes stand for main pathways based on enrichment analysis.

**Figure 5:**
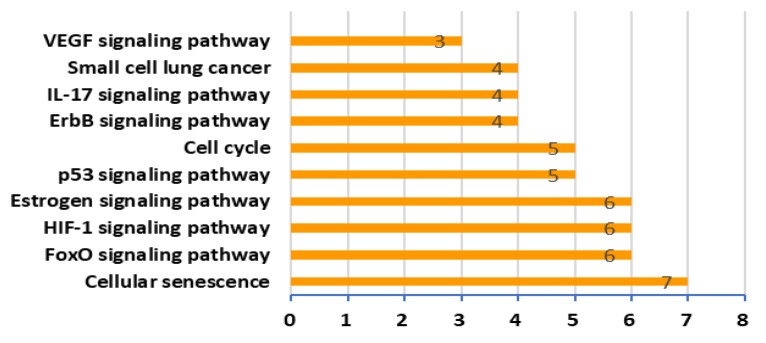
Main pathways enriched by major targets. The top 10 pathways selected to demonstrate the crucial biological actions of major targets. The abscissa stands for target counts in each pathway, and the ordinate stands for main pathways.

In order to investigate the biological effects of 41 targets of XQD for LC, 41 genes were inserted by ClueGO plug in Cytoscape. The target-pathway network graph (Figure 4) was obtained by setting a path that only showed *p* value ≤ 0.05 and in which the number of genes in the pathway was more than three. We analyzed the data and relevant biological processes for LC, choosing top ten remarkable significant pathways (Figure 5) according to the *p* value for further study. The first four pathways with the largest number of gene enrichment were cell senescence [50,51], FoxO signaling pathway [52,53], HIF-1 (hypoxia inducible factor) signaling pathway [54], and estrogen signaling pathway [55,56]. Thus pro-senescence therapies may represent a new treatment for lung cancer [57]. FoxO play a vital role in cell fate determination, and the subfamily is also considered to play a key role in cancer as a cancer inhibitor. In the process of apoptosis, FoxO participates in mitochondrial dependent and independent processes, triggering the expression of death receptor ligands such as Fas ligand, TNF apoptotic ligand and Bcl-XL [58]. HIF-1 signaling pathway inhibits cell viability and induces cell apoptosis [55]. The results indicate that HIF-1α signaling pathway plays an important role in the regulation of TNF-α-induced proliferation and metastasis of A549 cells in NSCLC [59]. In addition, there are five other pathways: p53 signaling pathway [60,61], cellcycle, ErbB signaling pathway [62], IL-17 signaling pathway [51,52,63,64], small cell lung cancer, vascular endothelial growth factor signaling pathway [65,66]. All of them are closely related to the occurrence of lung cancer, apoptosis of tumor cells, and immune function. Moreover, we drew a histogram of the 10 pathways screened, showing the number of genes enriched in each pathway.

### 3.6. Constructing Target-Immune Tissues and Cell Network

**Figure 6:**
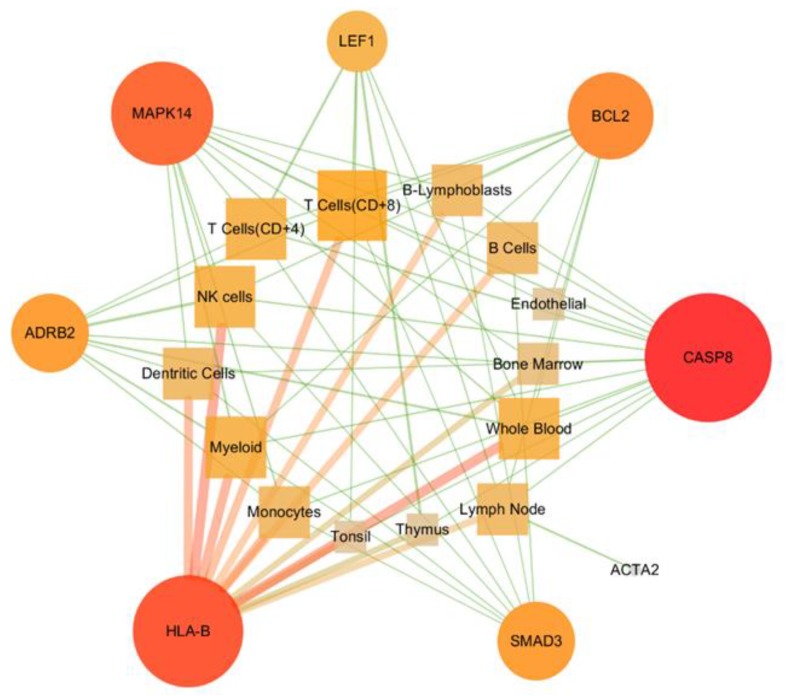
Target size and color settings were used to reflect Degree value size. The larger the shape and the darker the color of the node indicates that the target (gene) could play a role in the more immune tissues and cells.

The 41 LC genes affected by XQD were placed in the GeneCards database to search for the expression of targets (genes) in human tissues and cells. With the chi-square test, finally eight therapeutic targets for LC are significantly expressed in immune tissues and cells. The data of eight LC treatment targets and 14 immune tissues and cells were introduced into Cytoscape 3.7.1 to construct a network between target-immune (Figure 5) for further analysis. The size and color of the target were set to reflect the Degree size, and the thickness of the table was set to reflect the combine size. In this network, HLA-B, CASP8, and MAPK14 three targets of XQD, had the most obvious relationship with immune tissues and cells. From Figure 6 and Table 7, it can be seen that the expression level of HLA-B in 14 immune tissues and cells is significantly higher than that of the other seven targets. The expression of CASP8 in NK cells, CD8+T cells and CD4+T cells is significantly higher than that in the other 11 tissues and cells. MAPK14 also is more significantly expressed in 14 kinds of immune tissues and cells. Therefore, we concluded that the three targets of HLA-B, CASP8, and MAPK14 are the immunological targets of XQD for the treatment of LC.

## 4. Discussion

HLA-B is an important target of XQD in the treatment of LC and also an immunological target. HLA-B is one of the MHC I. MHC is the major histocompatibility complex of human, namely human Leukocyte Antigen HLA, which participates in antigen presentation, specifically recognizes TCR, and plays a key role in the activation of T cells. HLA-B belongs to HLA-I molecule, which is widely distributed on the surface of all nucleated cells and closely related to human immunity [67]. While complexing with antigenic peptides, HLA-B molecules initiate CD8+T cell responses via interaction with the T cell receptor (TCR) and coreceptor CD8 [68]. Similarly, the expression of HLA-B in cancer cells was helpful in activating the activation and proliferation of CD8+T cells [69].

CASP8 is a member of the caspase-cysteine protease family and plays an important role in the development of cancer [70]. It has been reported that 79% of NSCLC cell lines lack Caspase-8, about 35% of SCLC and 18% of bronchogenic carcinoma have promoter methylation of CASP8 [71]. Caspase-8 can activate Caspase downstream of almost all apoptotic cascades and induce apoptosis [72,73], as a caspase-dependent apoptotic pathway promoter, which has been extensively studied under the trigger of death receptor of TNF-R1 [74]. CASP8 has immuno-regulatory functions [75], which regulates T cell activation and proliferation [76], positive regulation of macrophage differentiation [77], and activation of natural killer cells [78].

MAPK14 activation could be a common response of most cancer cells. The family of p38 has 4 different isoforms (MAPK11/p38β, MAPK12/p38γ, MAPK13/p38δ, and MAPK14/p38α) [79], among which MAPK14 is the most abundant and widely expressed [80]. MAPK14 is an important apoptotic inducer, TNF-α, TGF-β and oxidative stress activate MAPK14 signaling pathway to induce apoptosis [81,82,83], and exert its anti-tumor effect [84]. Reactivation of the p38α MAPK pathway might be a useful therapy for LC [85].

Studies have shown that Fas-mediated increase in the activity of P38MAPK requires the participation of Caspase family members. P38MAPK is the downstream target of Caspase, thus causing target cell apoptosis [86]. Here are the mechanisms of P38MAPK in promoting apoptosis: 1) participating in Fas/FasL-mediated apoptosis [87]; 2) upregulating the expression of TNF-alpha; TNF can induce apoptosis by activating MAPKK upstream of p38MAPK, and ultimately activating p38MAPK [88]. Malignant proliferation of cells or the production of cancer cells are due to the dysfunction of cell proliferation and cell death regulation. Fas/FasL is the most important signal pathway involved in cell apoptosis [89], which is closely related to anti-cancer therapy [90].

**Figure 7:**
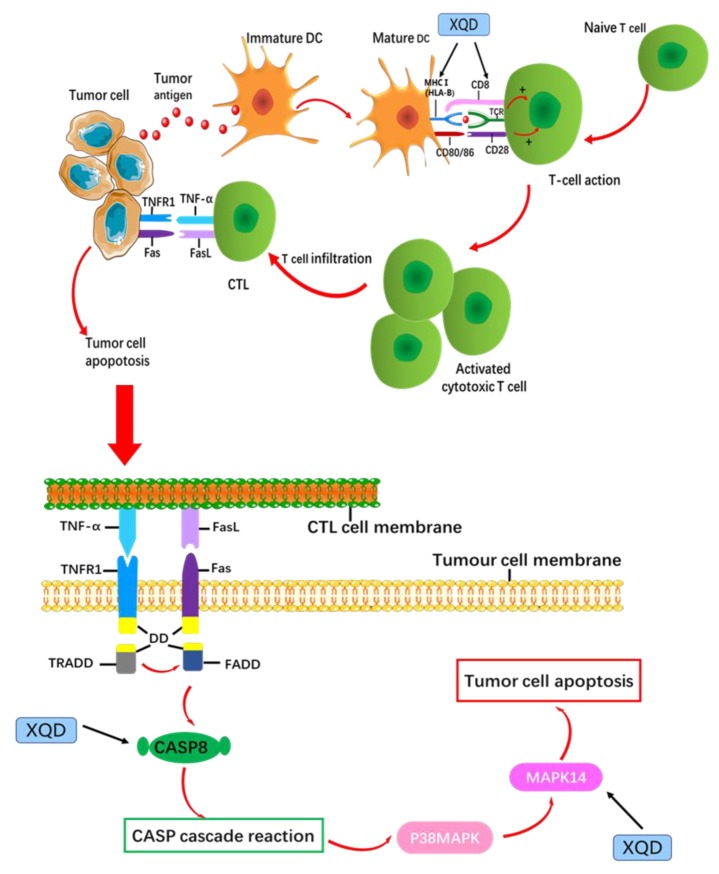
The target of XQD for LC therapy regulates immune-mediated tumor cell apoptosis. DC, dendritic cell; MHC, Major histocompatibility complex, TCR, T-cell receptor, CTL, Cytotoxic T cells, DD, Death domain, FADD, Fas-associated death domain, TRADD, TNF receptor associated death domain. HLA-B, CASP8 and MAPK14 were three therapeutic targets of XQD for LC.

XQD involves the biological process of tumor cell apoptosis induced by immune regulation (Figure 7). In stimulating the process of tumor cell apoptosis, XQD plays a role in HLA-B on dendritic cells (MHC I) of mature DC and CD8+T cells at the same time, to activate CD8+T cells in the immune response, to identify tumor antigen and kill tumor cells. After activation, CTL expresses FasL and TNF-α, binds to the Fas and TNFR1 on the surface of tumor cells, and generates FADD, which will conduct the apoptosis signal into the cell and perform CASP8 proteolytic activation, thus initiating the apoptosis process of tumor cells. The apoptotic signal was transmitted to MAPK14 to induce accelerated apoptosis.

As demonstrated in this study, XQD can play a vital role in the treatment of LC by enhancing immunity and mediating apoptosis of cancer cells. One of the characteristics of TCM is to seek the root of the disease. It advocates the idea of strengthening the body and eliminating pathogens in the treatment of diseases. In the mechanism of XQD in the treatment of LC, it can not only activate the function of immune cells and improve the immunity of patients, but also promote the apoptosis of cancer cells. From the molecular mechanism, the advantages of XQD in the treatment of LC are that it reinforces the healthy qi and eliminates the pathogenic factors. In the study of XQD, the practical application of network analysis method was described, and the results show that this method was an effective strategy for the modern research of TCM. The data of herbal ingredients involved in each formula cover a wide range and involve many targets, which in turn provides more directions for the research on the molecular mechanism of the therapeutic effect of TCM formula. Different TCM formulations have different components and targets. The network pharmacology method can better explain the different molecular mechanisms of different formulas for the treatment of various diseases. Although a lot of network pharmacology research has been done in the field of TCM, it has not shown the characteristic advantages of TCM therapy—Fu Zheng Fu Ben and strengthening the immunity. At present, research on LC rarely involve the effects of drugs on immune tissues and cells. This study aims to make up for this deficiency. Using a similar research analysis method, more cancers and tumors can be studied.

## 5. Conclusions

The study reveals the molecular mechanism of XQD in the treatment of LC from the perspective of network pharmacology. The aim of this study was to analyze the molecular mechanism of the effective components and targets in TCM prescriptions acting on lung cancer and immunity, and to understand the synergistic mechanism and characteristics of TCM from a more comprehensive perspective. The results show that this method can better explain the unique advantages of XQD in the treatment of LC.

While treating LC, XQD can activate (up-regulate) the function of immune cells, promote the apoptosis of tumor cells, and has an active anti-tumor immune effect. Therefore, it is necessary to give full play to the characteristics of TCM in the treatment of cancer: reinforce the healthy qi and eliminate the pathogenic factors. It provides a new idea for the current research of LC treatment, with the aim of treating LC while enhancing immunity.

## Figures and Tables

**Table 1:** Active ingredients and ADME parameters of Xia Qi Decoction (XQD).

ID	MOL ID	Molecule Name	OB/%	DL	Herb
ID001	MOL000359	sitosterol	36.91	0.75	CR, PR, FR, AC
ID002	MOL004328	naringenin	59.29	0.21	CR, LO
ID003	MOL005100	5,7-dihydroxy-2-(3-hydroxy-4-methoxyphenyl)chroman-4-one	47.74	0.27	CR
ID004	MOL005815	Citromitin	86.9	0.51	CR
ID005	MOL005828	nobiletin	61.67	0.52	CR
ID006	MOL001755	24-Ethylcholest-4-en-3-one	36.08	0.76	AT
ID007	MOL002670	Cavidine	35.64	0.81	AT
ID008	MOL002714	baicalein	33.52	0.21	AT
ID009	MOL002776	Baicalin	40.12	0.75	AT
ID010	MOL000358	beta-sitosterol	36.91	0.75	AT, FR, PR
ID011	MOL000449	Stigmasterol	43.83	0.76	AT, AC
ID012	MOL005030	gondoic acid	30.7	0.2	AT, AC
ID013	MOL000519	coniferin	31.11	0.32	AT
ID014	MOL006936	10,13-eicosadienoic	39.99	0.2	AT
ID015	MOL006937	12,13-epoxy-9-hydroxynonadeca-7,10-dienoic acid	42.15	0.24	AT
ID016	MOL006957	(3S,6S)-3-(benzyl)-6-(4-hydroxybenzyl)piperazine-2,5-quinone	46.89	0.27	AT
ID017	MOL003578	Cycloartenol	38.69	0.78	AT
ID018	MOL006967	beta-D-Ribofuranoside, xanthine-9	44.72	0.21	AT
ID019	MOL001910	11alpha,12alpha-epoxy-3beta-23-dihydroxy-30-norolean-20-en-28,12beta-olide	64.77	0.38	PR
ID020	MOL001918	paeoniflorgenone	87.59	0.37	PR
ID021	MOL001919	(3S,5R,8R,9R,10S,14S)-3,17-dihydroxy-4,4,8,10,14-pentamethyl-2,3,5,6,7,9-hexahydro-1H-cyclopenta[a]phenanthrene-15,16-dione	43.56	0.53	PR
ID022	MOL001921	Lactiflorin	49.12	0.8	PR
ID023	MOL001924	paeoniflorin	53.87	0.79	PR
ID024	MOL001925	paeoniflorin_qt	68.18	0.4	PR
ID025	MOL001928	albiflorin_qt	66.64	0.33	PR
ID026	MOL001930	benzoyl paeoniflorin	31.27	0.75	PR
ID027	MOL000211	Mairin	55.38	0.78	PR, LO, AC
ID028	MOL000422	kaempferol	41.88	0.24	PR
ID029	MOL000492	(+)-catechin	54.83	0.24	PR, AC
ID030	MOL004624	Longikaurin A	47.72	0.53	SC
ID031	MOL005317	Deoxyharringtonine	39.27	0.81	SC
ID032	MOL008956	Angeloylgomisin O	31.97	0.85	SC
ID033	MOL008957	Schizandrer B	30.71	0.83	SC
ID034	MOL008968	Gomisin-A	30.69	0.78	SC
ID035	MOL008974	Gomisin G	32.68	0.83	SC
ID036	MOL008978	Gomisin R	34.84	0.86	SC
ID037	MOL008992	Wuweizisu C	46.27	0.84	SC
ID038	MOL001749	ZINC03860434	43.59	0.35	FR
ID039	MOL004440	Peimisine	57.4	0.81	FR
ID040	MOL009027	Cyclopamine	55.42	0.82	FR
ID041	MOL009572	Chuanbeinone	41.07	0.71	FR
ID042	MOL009579	ent-(16S)-atisan-13,17-oxide	47.74	0.43	FR
ID043	MOL009586	isoverticine	48.23	0.67	FR
ID044	MOL009588	Korseveriline	35.16	0.68	FR
ID045	MOL009589	Korseverinine	53.51	0.71	FR
ID046	MOL009593	verticinone	60.07	0.67	FR
ID047	MOL009596	sinpemine A	46.96	0.71	FR
ID048	MOL009599	songbeinone	45.35	0.71	FR
ID049	MOL010921	estrone	53.56	0.32	AC
ID050	MOL010922	Diisooctyl succinate	31.62	0.23	AC
ID051	MOL002211	11,14-eicosadienoic acid	39.99	0.2	AC
ID052	MOL002372	(6Z,10E,14E,18E)-2,6,10,15,19,23-hexamethyltetracosa-2,6,10,14,18,22-hexaene	33.55	0.42	AC
ID053	MOL000953	CLR	37.87	0.68	AC
ID054	MOL002311	Glycyrol	90.78	0.67	AC, LO
ID055	MOL003410	Ziziphin_qt	66.95	0.62	AC
ID056	MOL004355	Spinasterol	42.98	0.76	AC
ID057	MOL004841	Licochalcone B	76.76	0.19	AC, LO
ID058	MOL004903	liquiritin	65.69	0.74	AC, LO
ID059	MOL004908	Glabridin	53.25	0.47	AC, LO
ID060	MOL005017	Phaseol	78.77	0.58	AC, LO
ID061	MOL007207	Machiline	79.64	0.24	AC
ID062	MOL012922	l-SPD	87.35	0.54	AC
ID063	MOL000273	(2R)-2-[(3S,5R,10S,13R,14R,16R,17R)-3,16-dihydroxy-4,4,10,13,14-pentamethyl-2,3,5,6,12,15,16,17-octahydro-1H-cyclopenta[a]phenanthren-17-yl]-6-methylhept-5-enoic acid	30.93	0.81	PC
ID064	MOL000275	trametenolic acid	38.71	0.8	PC
ID065	MOL000276	7,9(11)-dehydropachymic acid	35.11	0.81	PC
ID066	MOL000279	Cerevisterol	37.96	0.77	PC
ID067	MOL000280	(2R)-2-[(3S,5R,10S,13R,14R,16R,17R)-3,16-dihydroxy-4,4,10,13,14-pentamethyl-2,3,5,6,12,15,16,17-octahydro-1H-cyclopenta[a]phenanthren-17-yl]-5-isopropyl-hex-5-enoic acid	31.07	0.82	PC
ID068	MOL000282	ergosta-7,22E-dien-3beta-ol	43.51	0.72	PC
ID069	MOL000283	Ergosterol peroxide	40.36	0.81	PC
ID070	MOL000285	(2R)-2-[(5R,10S,13R,14R,16R,17R)-16-hydroxy-3-keto-4,4,10,13,14-pentamethyl-1,2,5,6,12,15,16,17-octahydrocyclopenta[a]phenanthren-17-yl]-5-isopropyl-hex-5-enoic acid	38.26	0.82	PC
ID071	MOL000287	3beta-Hydroxy-24-methylene-8-lanostene-21-oic acid	38.7	0.81	PC
ID072	MOL000289	pachymic acid	33.63	0.81	PC
ID073	MOL000290	Poricoic acid A	30.61	0.76	PC
ID074	MOL000291	Poricoic acid B	30.52	0.75	PC
ID075	MOL000292	poricoic acid C	38.15	0.75	PC
ID076	MOL000296	hederagenin	36.91	0.75	PC
ID077	MOL000300	dehydroeburicoic acid	44.17	0.83	PC
ID078	MOL005020	dehydroglyasperins C	53.82	0.37	LO
ID079	MOL005018	Xambioona	54.85	0.87	LO
ID080	MOL005012	Licoagroisoflavone	57.28	0.49	LO
ID081	MOL005007	Glyasperins M	72.67	0.59	LO
ID082	MOL005003	Licoagrocarpin	58.81	0.58	LO
ID083	MOL005001	Gancaonin H	50.1	0.78	LO
ID084	MOL005000	Gancaonin G	60.44	0.39	LO
ID085	MOL004993	8-prenylated eriodictyol	53.79	0.4	LO
ID086	MOL004990	7,2′,4′-trihydroxy-5-methoxy-3-arylcoumarin	83.71	0.27	LO
ID087	MOL004959	1-Methoxyphaseollidin	69.98	0.64	LO
ID088	MOL004941	(2R)-7-hydroxy-2-(4-hydroxyphenyl)chroman-4-one	71.12	0.18	LO
ID089	MOL004914	1,3-dihydroxy-8,9-dimethoxy-6-benzofurano[3,2-c]chromenone	62.9	0.53	LO
ID090	MOL004912	Glabrone	52.51	0.5	LO
ID091	MOL004910	Glabranin	52.9	0.31	LO
ID092	MOL004907	Glyzaglabrin	61.07	0.35	LO
ID093	MOL004904	licopyranocoumarin	80.36	0.65	LO
ID094	MOL004891	shinpterocarpin	80.3	0.73	LO
ID095	MOL004885	licoisoflavanone	52.47	0.54	LO
ID096	MOL004879	Glycyrin	52.61	0.47	LO
ID097	MOL004863	3-(3,4-dihydroxyphenyl)-5,7-dihydroxy-8-(3-methylbut-2-enyl)chromone	66.37	0.41	LO
ID098	MOL004856	Gancaonin A	51.08	0.4	LO
ID099	MOL004855	Licoricone	63.58	0.47	LO
ID100	MOL004849	3-(2,4-dihydroxyphenyl)-8-(1,1-dimethylprop-2-enyl)-7-hydroxy-5-methoxy-coumarin	59.62	0.43	LO
ID101	MOL004838	8-(6-hydroxy-2-benzofuranyl)-2,2-dimethyl-5-chromenol	58.44	0.38	LO
ID102	MOL004835	Glypallichalcone	61.6	0.19	LO
ID103	MOL004829	Glepidotin B	64.46	0.34	LO
ID104	MOL004824	(2S)-6-(2,4-dihydroxyphenyl)-2-(2-hydroxypropan-2-yl)-4-methoxy-2,3-dihydrofuro[3,2-g]chromen-7-one	60.25	0.63	LO
ID105	MOL004820	kanzonols W	50.48	0.52	LO
ID106	MOL004810	glyasperin F	75.84	0.54	LO
ID107	MOL004808	glyasperin B	65.22	0.44	LO
ID108	MOL003656	Lupiwighteone	51.64	0.37	LO
ID109	MOL001484	Inermine	75.18	0.54	LO
ID110	MOL000500	Vestitol	74.66	0.21	LO
ID111	MOL000392	formononetin	69.67	0.21	LO
ID112	MOL000239	Jaranol	50.83	0.29	LO

**Table 2:** The topological properties of key components.

Herb	MOL Name	MOL ID	Degree
FR PR AT	Beta-sitosterol	MOL000358	49
PR	Kaempferol	MOL000422	44
AC	L-SPD	MOL012922	44
AT	Cavidine	MOL002670	43
AC AT	Stigmasterol	MOL000449	39
AC	Estrone	MOL010921	34
AC	Machiline	MOL007207	32
LO	Shinpterocarpin	MOL004891	30
LO	Formononetin	MOL000392	30
LO	Naringenin	MOL004328	29
LO CR	1-Methoxyphaseollidin	MOL004959	28
LO	Licoagrocarpin	MOL005003	28

**Table 3:** The Topological Properties of Targets.

Target ID	Gene Name	Protein Name	Degree
P10275	AR	Androgen receptor	83
P03372	ESR1	Estrogen receptor	74
P00918	CA2	Carbonic anhydrase 2	60
P37231	PPARG	Peroxisome proliferator-activated receptor gamma	56
P35354	PTGS2	Prostaglandin G/H synthase 2	56
P35228	NOS2	Nitric oxide synthase, inducible	53
P00734	F2	Prothrombin	49
P27487	DPP4	Dipeptidyl peptidase 4	49
Q92731	ESR2	Estrogen receptor beta	47
Q07785	CRK2	Cell division control protein 2 homolog	45
P49841	GSK3B	Glycogen synthase kinase-3 beta	46
O14757	CHEK1	Serine/threonine-protein kinase Chk1	45
P18031	PTPN1	Tyrosine-protein phosphatase non-receptor type 1	43
Q16539	MAPK14	Mitogen-activated protein kinase 14	39
P07900	HSP90AA1	Heat shock protein HSP 90-alpha	38
P08238	HSP90AB1	Heat shock protein HSP 90-beta	37
P23219	PTGS1	Prostaglandin G/H synthase 1	32
P00742	F10	Coagulation factor X	31
Q14524	SCN5A	Sodium channel protein type 5 subunit alpha	30

**Table 4:** Key components of XQD for lung cancer (LC) treatment.

Herb	MOL ID	MOL Name	Degree
FR PR AT	MOL000358	Beta-sitosterol	17
PR	MOL000422	Kaempferol	14
AT	MOL002670	Cavidine	13
CR LO	MOL004328	Naringenin	12
LO	MOL000392	Formononetin	11
AC	MOL012922	l-SPD	11
AT	MOL002714	Baicalein	10
AT AC	MOL000449	Stigmasterol	10
LO	MOL005003	Licoagrocarpin	10
LO	MOL004959	1-Methoxyphaseollidin	10
LO AC	MOL004908	Glabridin	10
LO	MOL004891	Shinpterocarpin	10
AC	MOL004841	Licochalcone B	10
LO	MOL001484	Inermine	10
AC	MOL010921	Estrone	10
AC PR	MOL000492	(+)-catechin	9
CR	MOL005828	Nobiletin	9
LO AC	MOL005017	Phaseol	9
LO	MOL005000	Gancaonin G	9
LO	MOL004941	(2R)-7-hydroxy-2-(4-Hydroxyphenyl)chroman-4-one	9
LO	MOL004849	3-(2,4-dihydroxyphenyl)-8-(1,1-Dimethylprop-2-enyl)-7-hydroxy-5-methoxy-coumarin	9
LO	MOL004835	Glypallichalcone	9
LO	MOL004829	Glepidotin B	9
LO	MOL004824	(2S)-6-(2,4-dihydroxyphenyl)-2-(2-hydroxypropan-2-yl)-4-methoxy-2,3-dihydrofuro[3,2-g]chromen-7-one	9
AC	MOL007207	Machiline	9

**Table 5:** Direct Action of 14 LC Therapeutic targets.

Types of Action	Degree	Uniprot ID	Gene Name
Direct action	75	P03372	ESR1
Direct action	45	O14757	CHEK1
Direct action	40	Q16539	MAPK14
Direct action	28	P07550	ADRB2
Direct action	16	P29474	NOS3
Direct action	14	P35968	KDR
Direct action	11	P35372	OPRM1
Direct action	6	P10415	BCL2
Direct action	3	P08588	ADRB1
Direct action	1	P27169	PON1
Direct action	1	P14416	DRD2
Direct action	1	P05164	MPO
Direct action	1	P03956	MMP1
Direct action	1	P01130	LDLR

**Table 6:** Indirect action of 27 LC Therapeutic Targets.

Types of Action	Degree	Uniprot ID	Gene Name
Indirect action	5	P60568	IL2
Indirect action	5	O14746	TERT
Indirect action	4	Q9UJU2	LEF1
Indirect action	3	Q03468	ERCC6
Indirect action	3	P69905	HBA2
Indirect action	2	P62736	ACTA2
Indirect action	2	P16473	TSHR
Indirect action	2	P04035	HMGCR
Indirect action	2	P02741	CRP
Indirect action	1	Q9UBP4	DKK3
Indirect action	1	Q9H3N8	HRH4
Indirect action	1	Q9H3D4	TP63
Indirect action	1	Q14790	CASP8
Indirect action	1	Q12988	HSPB3
Indirect action	1	P84022	SMAD3
Indirect action	1	P57071	PRDM15
Indirect action	1	P42345	MTOR
Indirect action	1	P35568	IRS1
Indirect action	1	P30874	SSTR2
Indirect action	1	P25391	LAMA1
Indirect action	1	P18074	ERCC2
Indirect action	1	P08183	ABCB1
Indirect action	1	P05556	ITGB1
Indirect action	1	P04626	ERBB2
Indirect action	1	P01889	HLA-B
Indirect action	1	P00519	ABL1
Indirect action	1	O60469	DSCAM

**Table 7:** Expression of 8 LC treatment targets in 14 immune tissues and cells.

Name	HLA-B	CASP8	MAPK14	SMAD3	ACTA2	ADRB2	BCL2	LEF1
**Bone Marrow**	268	6	5	4	8	10	5	4
**Whole Blood**	689	12	13	4	9	25	6	9
**Lymph Node**	316	8	3	4	23	3	6	8
**Thymus**	229	6	3	4	17	2	4	35
**Tonsil**	302	6	3	4	11	3	5	6
**Myeloid**	630	11	21	5	9	22	6	4
**Monocytes**	577	9	13	5	8	9	5	4
**Dentritic Cells**	558	8	10	6	6	6	5	4
**NK cells**	716	13	10	5	8	41	7	5
**T Cells(CD+4)**	514	16	7	7	6	5	11	30
**T Cells(CD+8)**	512	15	7	5	7	14	8	27
**B-Lymphoblasts**	410	7	8	5	12	3	9	4
**B Cells**	411	8	5	8	7	3	11	4
**Endothelial**	154	6	5	4	5	3	6	4
